# Eliminating computed tomography imaging artifacts through 3D printed radiotherapy head supports

**DOI:** 10.1002/acm2.13027

**Published:** 2020-09-29

**Authors:** Stéphane Bedwani, Danis Blais, David Roberge, François DeBlois

**Affiliations:** ^1^ Département de radio‐oncologie Centre hospitalier de l'Université de Montréal (CHUM) Montréal QC Canada

**Keywords:** 3D printing, CT artifact, Head CT scan, Immobilization

## Abstract

**Purpose:**

The geometry of an immobilization device such as a headrest can cause undesired computed tomography (CT) artifacts that may affect both volume definition and dosimetry in radiotherapy of the brain. The purpose of this work was to reduce CT artifacts caused by a standard hard plastic hollow radiotherapy headrest. This was to be achieved through design and prototyping of a custom‐made head support.

**Methods:**

A series of CT scans were acquired of both a water phantom and an anthropomorphic head phantom which were resting on custom‐made three‐dimensional (3D) printed supports. All custom‐made supports were made of polylactic acid (PLA) plastic filament and printed by fused deposition modeling (FDM) 3D printing technology. Initial designs were studied with a water phantom using a simplified support with straight and curved shapes both at the edges and as infill patterns. Imaging of a 3D printed clinical prototype was then compared to our standard headrest using an anthropomorphic head phantom.

**Results:**

The presence of dark streaks inside both phantoms was seen on the CT images for headrests involving supports with straight shapes at the edges or as infill patterns. Such artifacts were ascribed to the exponential edge‐gradient effect (EEGE). No such artifact was observed when the support was designed with a combination of curved edges and infill patterns.

**Conclusion:**

When developing immobilization accessories for use in CT scanners, more attention could be paid to artifact attenuating design elements. This work illustrates the usefulness of 3D printing in prototyping radiotherapy accessories and solving concrete clinical problems.

## 1 INTRODUCTION

While planning radiotherapy for brain cancer treatment in our clinic, computed tomography (CT) images exhibit dark streak artifacts that can interfere with detection and delineation of structures of interest. Inspection of these images reveals that most streaks appear to radiate from the edge of the plastic headrest used during the CT scan.

The signature of this artifact is similar to a specific case of partial volume artifact also referred as the exponential edge‐gradient effect (EEGE).^1^ The latter is partly explained by a slight underestimation of the linear attenuation coefficient when the boundary between two media is parallel to the X‐ray beam of the CT scanner. If this border extends as a straight line, then the underestimation will be summed and back‐projected, resulting in dark streaks in the CT images.^2^

The main objective of this work was to develop a new headrest design to prevent EEGE artifacts. The first part of the study was carried out on a water phantom to reproduce the clinical conditions causing the artifact. A set of simplified phantom supports were three‐dimensional (3D) printed using different shapes in order to influence the presence of artifacts. This allowed us to test the theoretical concepts underlying the headrest design and assess the impact of the infill pattern required for 3D printing. The second part of the study was carried out with an anthropomorphic head phantom to compare the new headrest design with the standard clinical one.

## 2 MATERIALS AND METHODS

**Fig. 1:**
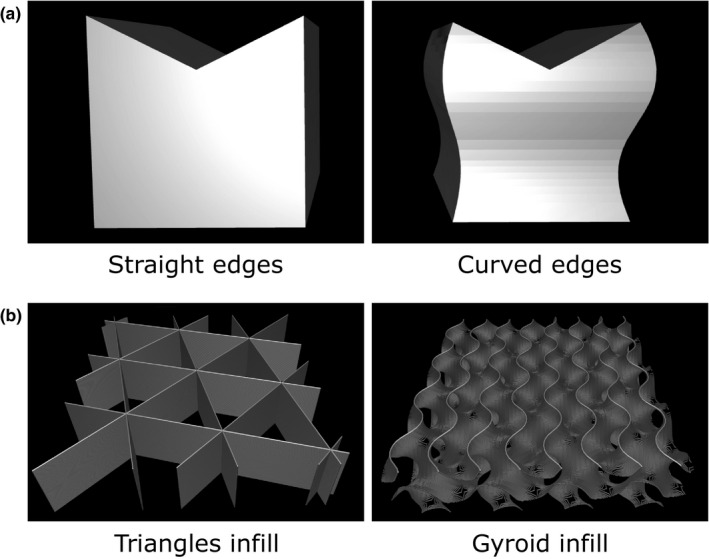
Simplified support models and infill patterns for three‐dimensional (3D) printing. (a) The support with straight edges (left) and curved edges (right) are both sculpted from a cube of dimensions (40 × 40 × 40) mm^3^. (b) Infill patterns, such as triangles (left) and gyroid (right), are required for 3D printing.

Two designs of a simplified support were made starting from a cube of dimension (40 × 40 × 40) mm^3^. The first support attempted to reproduce the properties of a clinical headrest model using straight lateral sides. A V‐shaped opening is added at the top to support a cylindrical phantom. The second support reproduces the dimensions of the previous model except for its four lateral S‐shaped sides. A 3D representation of the "straight" and "curved" support is illustrated in Fig. 1(a). Both designs were made using Fusion 360 (Autodesk, Inc., San Rafael, California, USA).

**Fig. 2:**
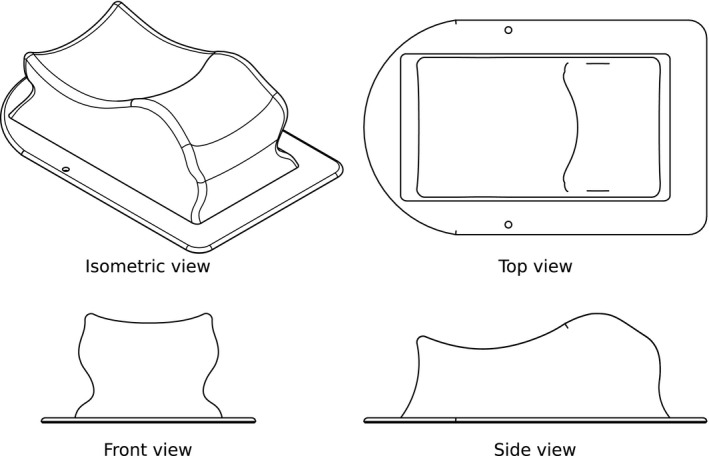
Technical drawing of the artifact‐free headrest. The isometric, front, side, and top views show the headrest model of maximum dimensions (280 × 175 × 88) mm^3^.

The clinical headrest model used in this study is the Silverman size F (Civco Medical Solutions, Rotterdam, Netherlands). The proposed design reproduces the same height at the nape of the neck, but the head tilt has been modified for comfort. The lateral sides of the headrest have been designed with curved surfaces. Its technical drawing is presented in Fig. 2, which was created using Fusion 360 (Autodesk, Inc.).

Both the simplified supports and the redesigned headrest were printed using a fused deposition modeling (FDM) Ultimaker S5 printer (Ultimaker, Utrecht, Netherlands) and polylactic acid (PLA) filament. The Cura software (Ultimaker) was used to convert 3D object models from stereolithography (.stl) files to specific instructions for the 3D printer. The printing parameters were set with a nozzle diameter of 0.4 mm and a layer thickness of 0.15 mm. Each simplified support model is printed with an infill density of 20% using either a "triangles" or "gyroid" pattern, as shown in Fig. 1(b). The redesigned headrest model is printed with an infill density of 10% using the gyroid pattern. A list of the printing parameters is provided in Table 1.

**Table 1:** Printing parameters used for the custom‐made head support.

Parameter	Value
Nozzle diameter	0.4 mm
Infill density	10%
Layer thickness	0.15 mm
Shell thickness (top/bottom/wall)	1.0 mm
Print speed	
Outer wall	20 mm/s
Inner wall	30 mm/s
Top and bottom layers	30 mm/s
Infill	70 mm/s
Temperature	
Extrusion	200°C
Build plate	60°C

**Fig. 3:**
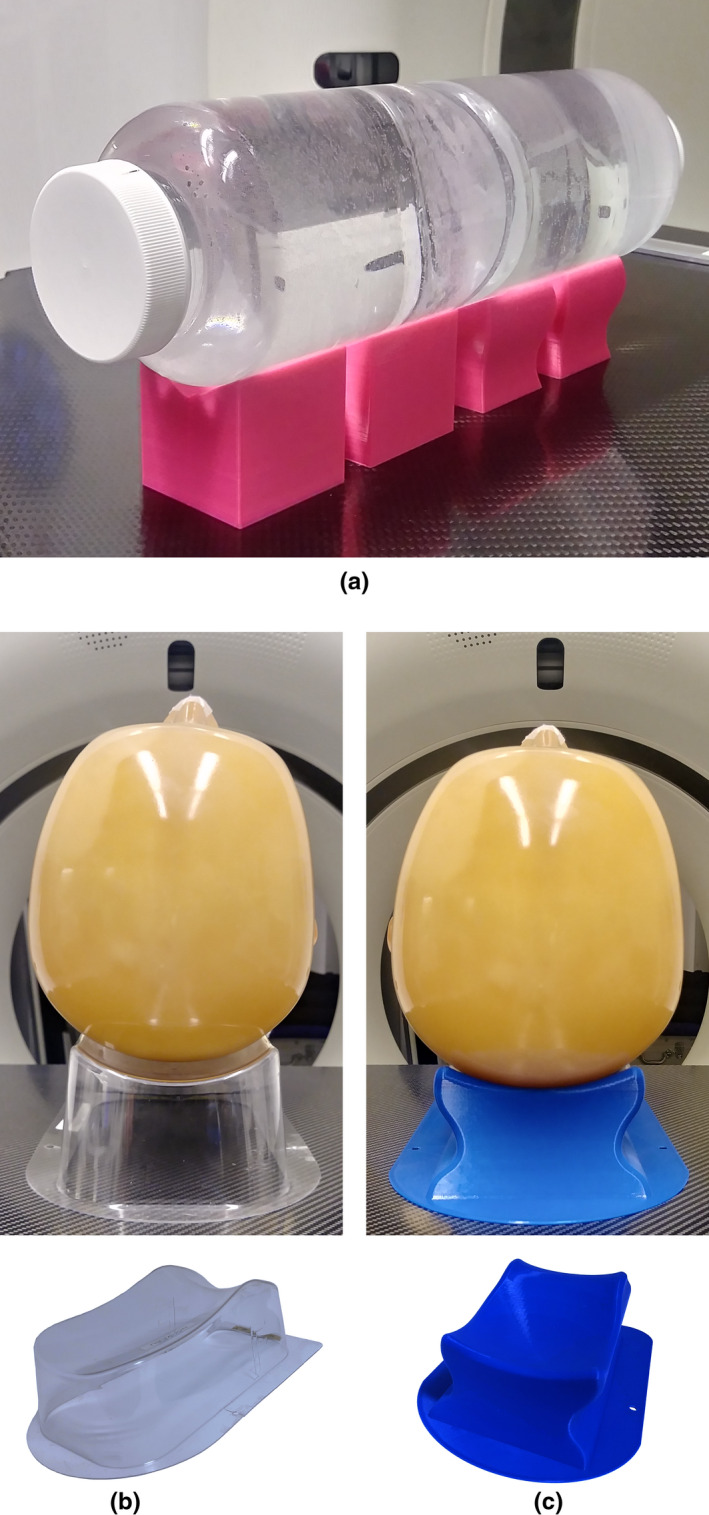
Phantoms with their supports. (a) The water phantom rests on four variations of the simplified support. The head phantom lies on (b) the clinical headrest and (c) the custom‐made headrest.

A 400 mL polyethylene terephthalate container (72 mm diameter) is used as a water phantom. A head of an anthropomorphic phantom PBU‐60 (Kyoto Kagaku Co., Ltd., Kyoto, Japan) is used for the comparison of headrest models. For each combination of phantom and support, Fig. 3 shows the experimental setup.

The CT scan acquisitions were carried out on a SOMATOM Definition Flash (Siemens Healthineers AG, Erlangen, Germany), and have used a standard head protocol with a tube voltage of 120 kV, an exposure of 465 mAs, a slice thickness of 1 mm, a pitch of 0.55, and the H41s reconstruction kernel. The field of view (FOV) was 150 mm for scans with the water phantom and 400 mm with the head phantom. The rigid registration (6D) between the CT images of the head phantom with and without headrest was performed on a MIMvista workstation (MIM Software Inc., Cleveland, Ohio, USA).

## 3 RESULTS

**Fig. 4:**
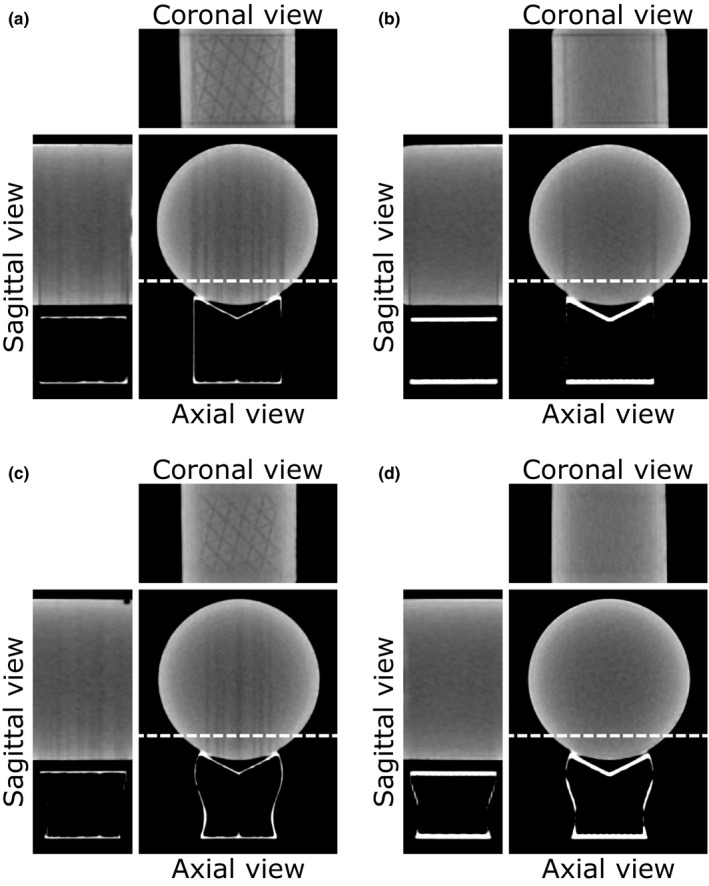
Computed tomography images of the water phantom on the four variations of a simplified support. The axial and sagittal views are centered on the support, while the location of the coronal view is indicated by the dashed line. The window center is 50 HU and the window width is 220 HU. The four variants of the support consist of the combination of the shape of side walls and infill patterns, such as (a) straight and triangles, (b) straight and gyroid, (c) curved and triangles, and (d) curved and gyroid.

The CT images obtained with the simplified supports and the water phantom are presented in Fig. 4. Figures 4(a) and 4(b) show vertical dark streaks emanating from each side of the support in both axial and sagittal views. A square‐shaped artifact appears in the coronal view. Figures 4(a) and 4(c) show extra dark streaks this time emanating from the inside of the support in both axial and sagittal views, while the triangular pattern of the printed infill appears in the coronal view. Figure 4(d) exhibits artifact‐free CT images achieved with the combination of the curved edge support and the gyroid infill pattern.

**Fig. 5:**
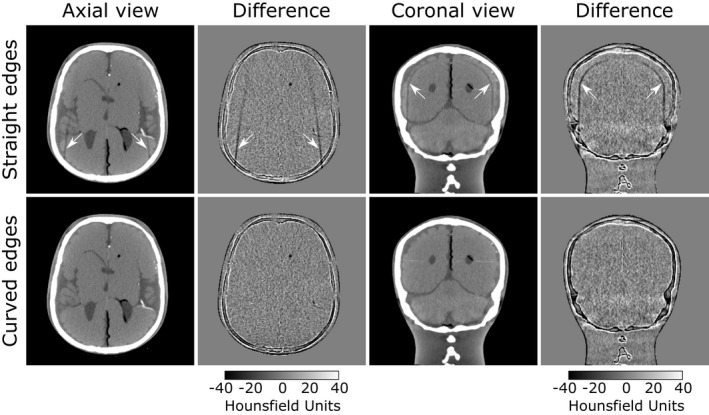
Computed tomography images of the head phantom on two headrests models. The top row shows the data obtained with the clinical headrest and the bottom row with the custom‐made one. The window center is 50 HU and the window width is 220 HU. Computed tomography volumes are registered on an acquisition without headrest, also used to generate the difference maps with a greyscale bar expressed in HU. The arrows point to the artifacts introduced by the presence of the headrest, visible on the axial and coronal views.

The CT images obtained with the headrests and the anthropomorphic head phantom are presented in Fig. 5. Artifacts with the standard support are clearly visible on the axial and coronal slices, while CT images with the new support design show none. The difference maps are obtained by subtracting the CT volume without headrest from the registered CT volume with headrest.

## 4 DISCUSSION

The origin of the artifact is studied on the water phantom with a set of simplified supports to determine which design features have to be included in our custom‐made headrest. Computed tomography images on the water phantom shown in Fig. 4 allows an EEGE‐type artifact to be attributed to the presence of straight structures in the geometry of the support, both on the lateral sides and infill pattern. One way to avoid this type of artifact is to replace the side walls of the support with curved shapes. Three‐dimensional printing also brings a new set of constraints, notably with the use of an infill. The choice of an infill pattern with rounded shapes, such as the gyroid, is indicated to avoid introducing new EEGE‐type artifacts. Figure 5 reflects the clinical application of these principles by demonstrating that a headrest with rounded shapes yields artifact‐free images. The difference maps highlight the presence or absence of such artifacts, but also reflect small unrelated registration errors at the edges of bone structures.

## 5 CONCLUSION

A 3D printed headrest has been designed avoiding straight walls and infill patterns, which were found to be causing undesired streak artifacts on CT scans of the head. This work also provided another example of 3D printing benefiting the clinical development of medical accessories. When developing immobilization accessories for use in CT scanners, more attention could be paid to artifact attenuating design elements. Future works will include developing a web‐based platform to share our 3D designs to the radiation oncology community.

## CONFLICTS OF INTEREST

No conflicts of interest.
